# Supplementary figures and images for: Accounting for eXentricities: Analysis of the X Chromosome in GWAS Reveals X-Linked Genes Implicated in Autoimmune Diseases
Source: PLoS One. 2014 Dec 5;9(12):e113684. doi: 10.1371/journal.pone.0113684 (PMC4257614; doi:10.1371/journal.pone.0113684)

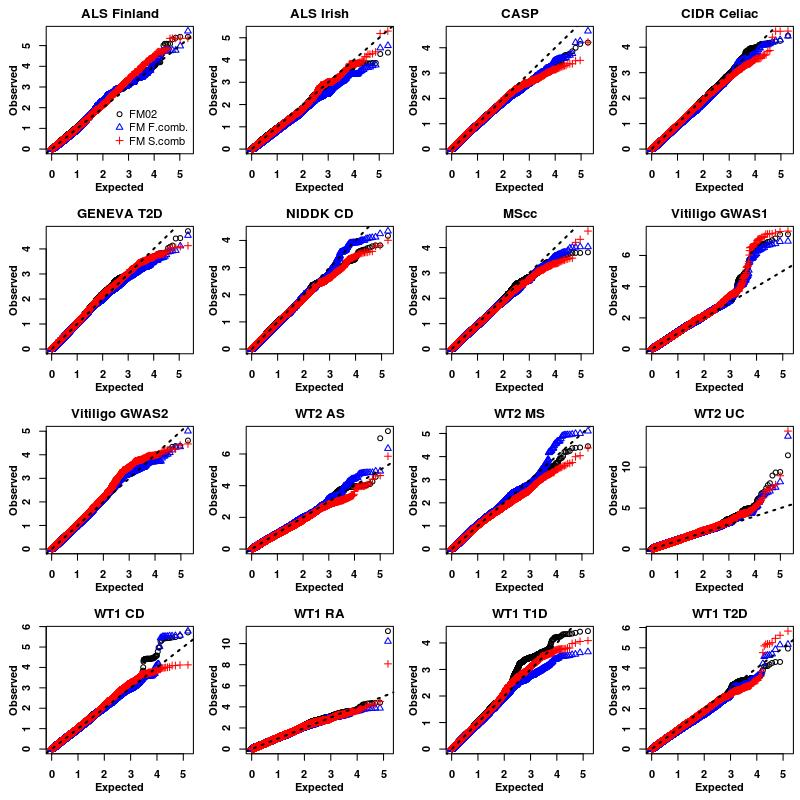

Supplement: Figure S1 — QQ-plots for single marker association tests. Blue triangles denote association p-values for the FMF.comb test, red crosses denote p-values for the FMS.comb, while the black points denote association p-values for the FM02 test. P-values are plotted on a log scale. Respective genomic inflation factors are summarized in Table S1. (TIFF) [file pone.0113684.s001.tiff]

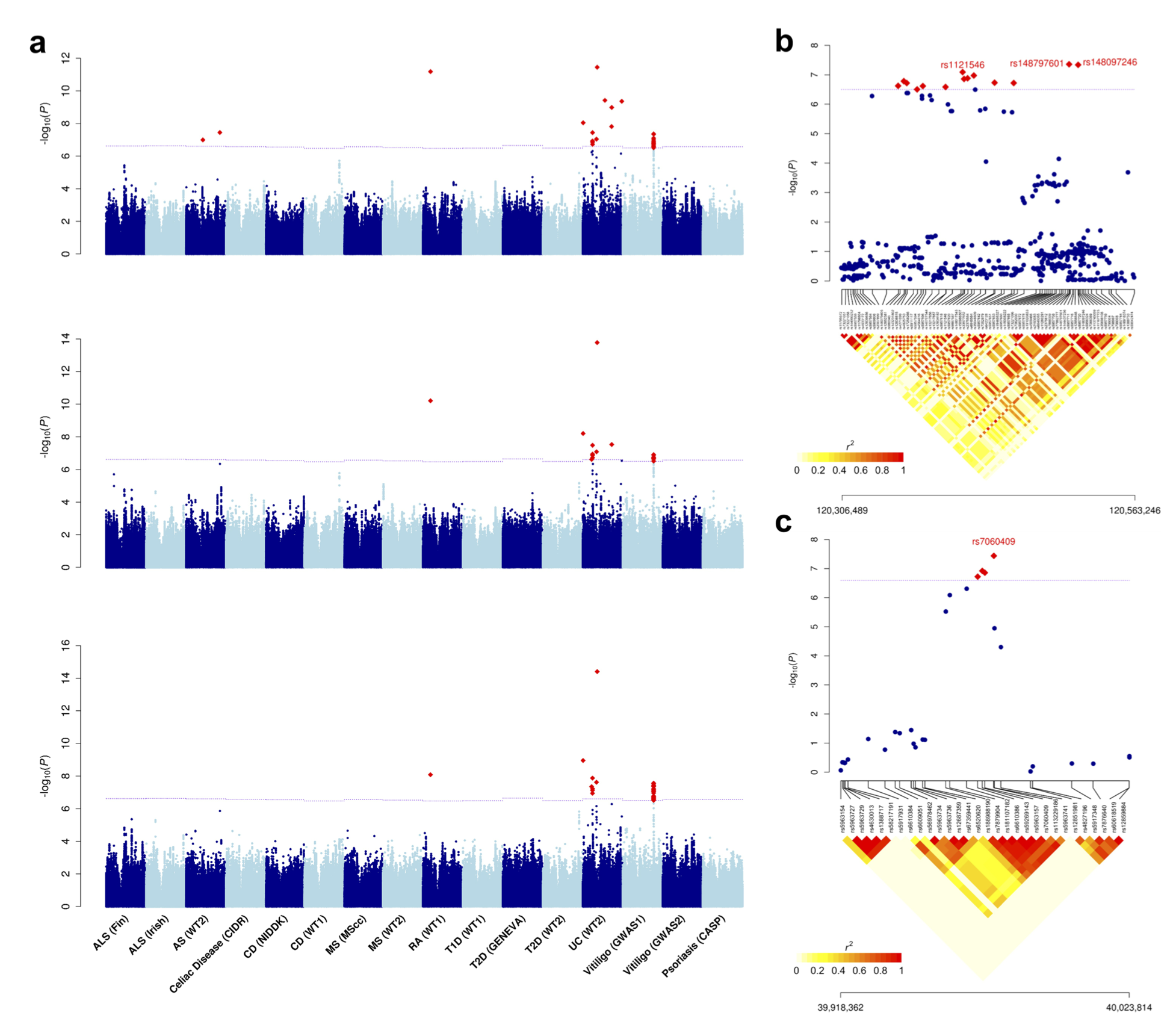

Supplement: Figure S2 — Significant SNP associations. (a) A Manhattan plot of the nominal p-values for the FM02 (upper), FMF.comb (middle), and FMS.comb (lower) tests of association for chromosome X SNPs in the 16 datasets. The dotted purple lines correspond to the X-chromosome-wide significance threshold for each dataset. The significant associations are shown as red diamonds. (b–c) Regional association plots of the association results of the FM02 test and LD for (b) Vitiligo GWAS1 dataset and (c) WT2 UC dataset. LD structure was plotted using a revised version of the snp.plotter software [165]. Due to the large number of SNPs in the associated region of Vitiligo GWAS1, only 1 in every 10 of the non-significantly associated SNPs is shown. We focus on regions presented in (b) and (c) since they show the typical LD peaks around significant association signals. (TIFF) [file pone.0113684.s002.tiff]

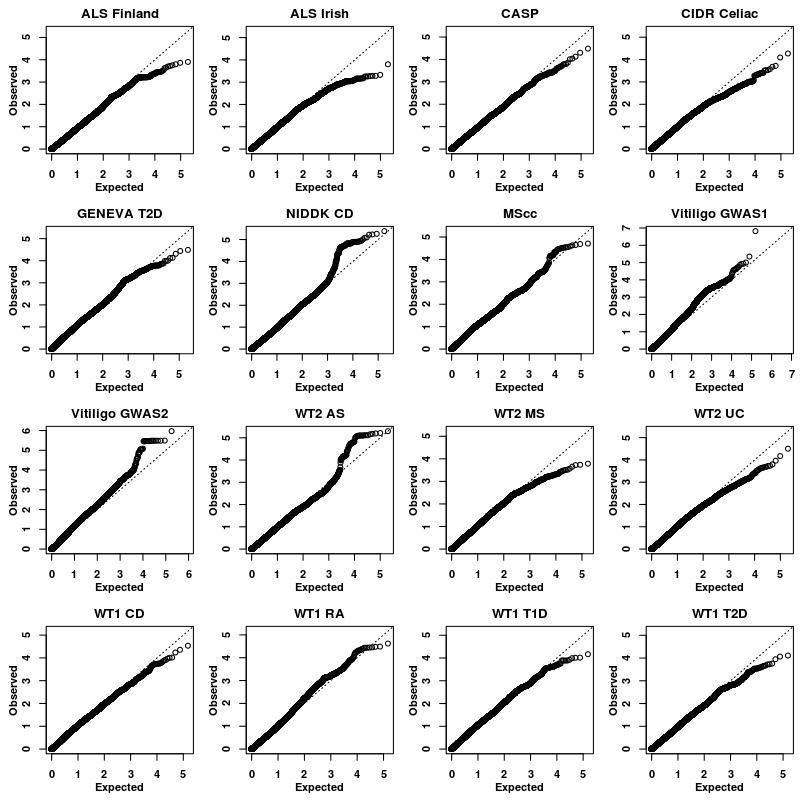

Supplement: Figure S3 — QQ-plots for test of sex-differentiated effect size. Similar to Figure S1, except that p-values are for the test of differential effect size between males and females. Respective genomic inflation factors are summarized in Table S1. (TIFF) [file pone.0113684.s003.tiff]

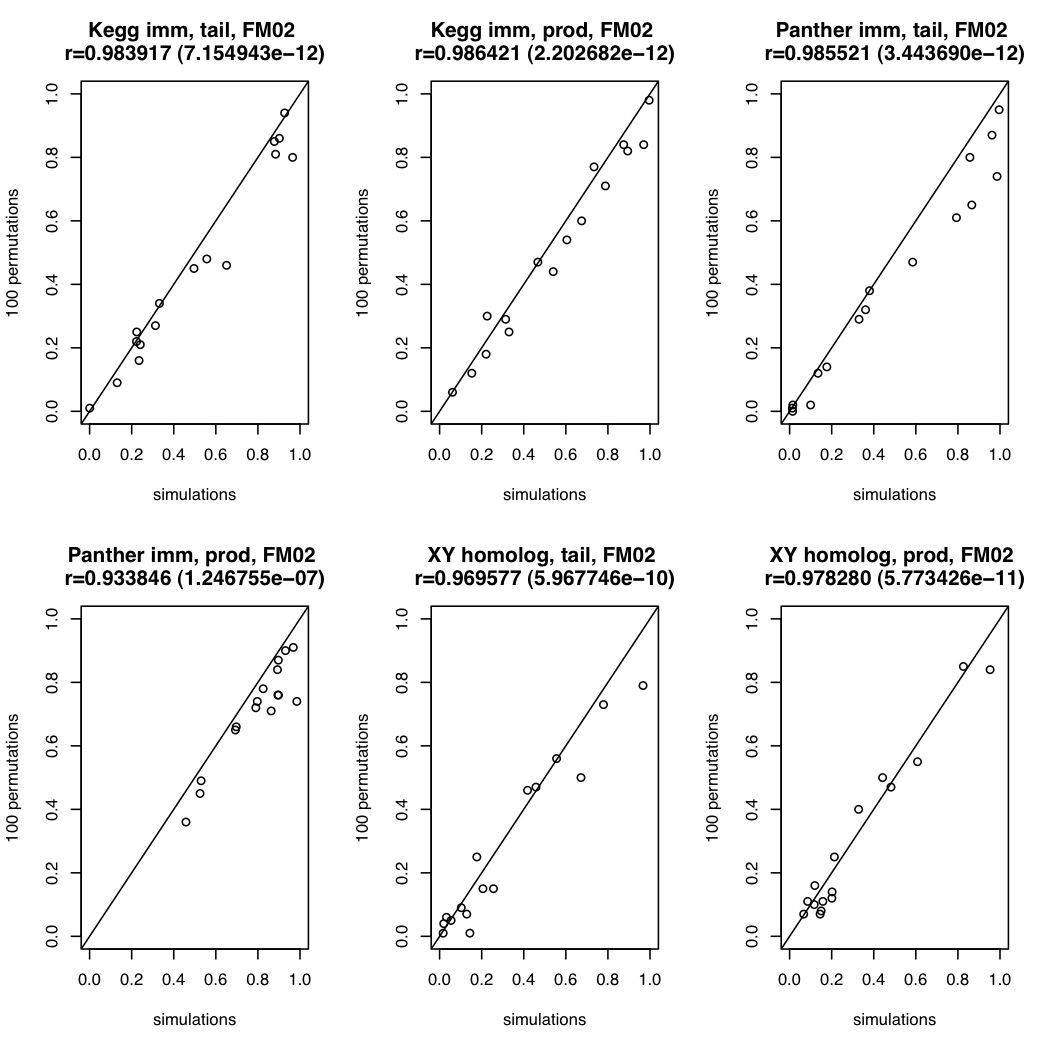

Supplement: Figure S4 — Simulation versus permutation derived p-values for gene-set tests for FM02. Comparison between simulation derived (x-axis) and permutation derived (y-axis) p-values for the gene-set association analysis using the FM02 test statistic. r represents Pearson's correlation coefficient and the significance of the correlation is indicated in parentheses in scientific notation. (TIFF) [file pone.0113684.s004.tiff]

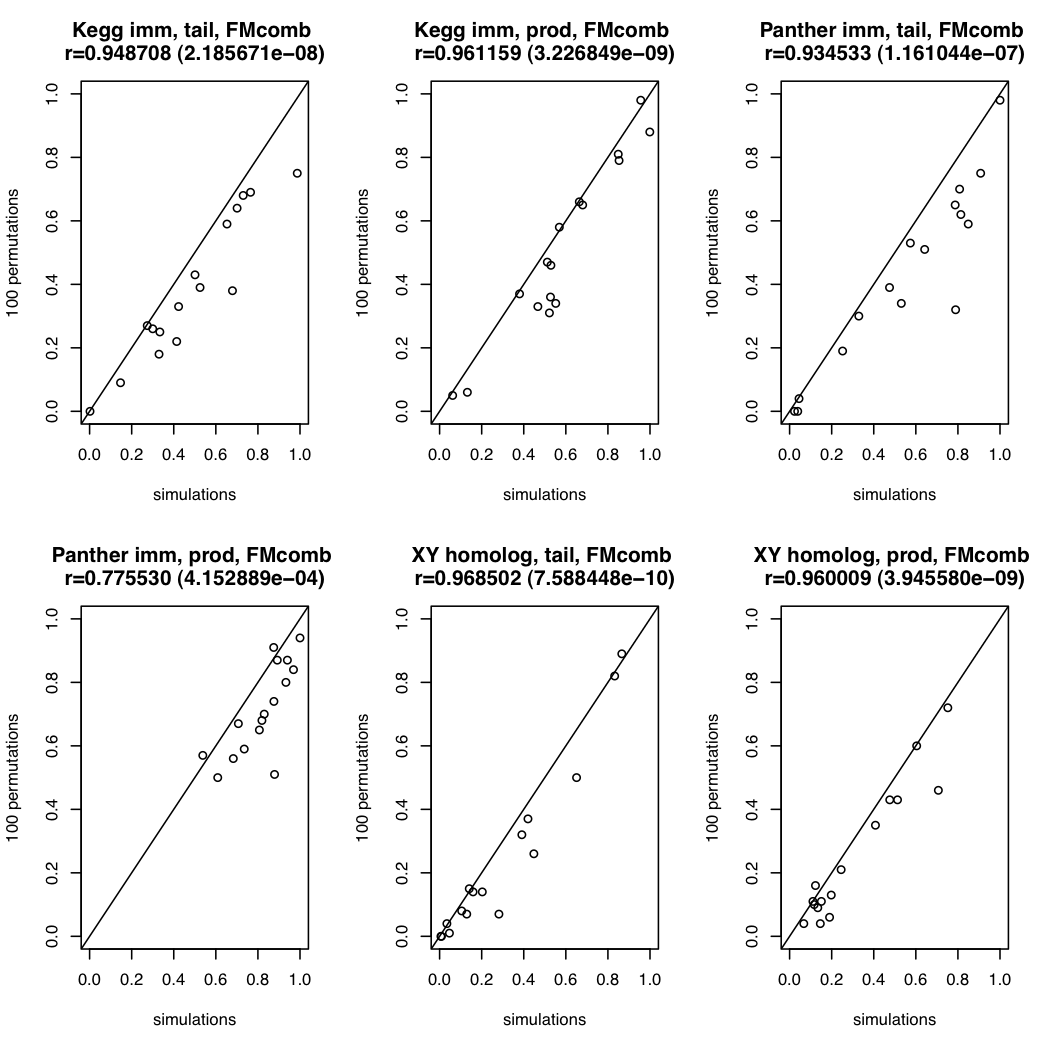

Supplement: Figure S5 — Simulation versus permutation derived p-values for gene-set tests for FMF.comb. Similar to Figure S4 except for considering the FMF.comb test statistic. (TIFF) [file pone.0113684.s005.tiff]
