# Supplementary material for: Accounting for eXentricities: Analysis of the X Chromosome in GWAS Reveals X-Linked Genes Implicated in Autoimmune Diseases
Source: PLoS One. 2014 Dec 5;9(12):e113684. doi: 10.1371/journal.pone.0113684 (PMC4257614; doi:10.1371/journal.pone.0113684)
Supplement: Table S1 — Genomic inflation factors were calculated from the observed p-values in the various tests. No inflation factor exceeds 1.14. Together with the respective QQ-plots (Figures S1 and S3) these results suggest little to no inflation in the observed SNP-level p-values. (DOC) [file pone.0113684.s006.doc]

| **Dataset** | **FM_02_** | **FM_F.comb_** | **FM_S.comb_** | **Sex difference** |
| --- | --- | --- | --- | --- |
| ALS Finland | 1.11 | 1.09 | 1.06 | 1.06 |
| ALS Irish | 0.92 | 0.83 | 0.90 | 0.87 |
| Psoriasis CASP | 0.96 | 0.95 | 0.96 | 1.00 |
| Celiac Disease CIDR | 0.94 | 0.91 | 0.94 | 0.92 |
| T2D GENEVA | 1.08 | 1.05 | 1.07 | 1.02 |
| CD NIDDK | 0.95 | 0.94 | 0.93 | 0.97 |
| MS Case Control | 0.96 | 0.95 | 0.93 | 0.95 |
| Vitiligo GWAS1 | 1.13 | 1.12 | 1.12 | 1.09 |
| Vitiligo GWAS2 | 1.10 | 1.07 | 1.10 | 0.99 |
| AS WT2 | 0.93 | 0.98 | 0.91 | 1.02 |
| MS WT2 | 1.00 | 1.05 | 0.94 | 1.04 |
| UC WT2 | 1.14 | 1.08 | 1.12 | 1.02 |
| CD WT1 | 1.07 | 1.03 | 1.06 | 1.00 |
| RA WT1 | 1.10 | 1.03 | 1.07 | 1.06 |
| T1D WT1 | 1.06 | 0.97 | 1.03 | 0.96 |
| T2D WT1 | 0.91 | 0.88 | 0.93 | 0.91 |
