# Supplementary material for: Accounting for eXentricities: Analysis of the X Chromosome in GWAS Reveals X-Linked Genes Implicated in Autoimmune Diseases
Source: PLoS One. 2014 Dec 5;9(12):e113684. doi: 10.1371/journal.pone.0113684 (PMC4257614; doi:10.1371/journal.pone.0113684)
Supplement: Table S2 — All significant associations (adjusted P<0.05) as observed in Figure S2. P-values are Bonferroni adjusted for the number of SNPs tested (Table 1). (DOC) [file pone.0113684.s007.doc]

| **Dataset** | **SNP** | **FM_02_ adjusted** | **FM_F.comb_ adjusted** | **FM_S.comb_ adjusted** |
| --- | --- | --- | --- | --- |
| **Vitiligo GWAS1** | rs2007899 | 8.24x10^-02^ | 1.42x10^-01^ | 2.90x10^-02^ |
|  | rs12852381 | 3.72x10^-02^ | 6.51x10^-02^ | 1.34x10^-02^ |
|  | rs143231802 | 2.58x10^-02^ | 3.62x10^-02^ | 6.47x10^-03^ |
|  | rs4271099 | 2.99x10^-02^ | 5.27x10^-02^ | 1.09x10^-02^ |
|  | rs4335270 | 6.52x10^-02^ | 1.23x10^-01^ | 2.67x10^-02^ |
|  | rs4480250 | 6.52x10^-02^ | 1.23x10^-01^ | 2.67x10^-02^ |
|  | rs17258266 | 4.90x10^-02^ | 7.55x10^-02^ | 1.38x10^-02^ |
|  | rs4300122 | 8.14x10^-02^ | 1.61x10^-01^ | 3.62x10^-02^ |
|  | rs5957594 | 3.77x10^-02^ | 7.27x10^-02^ | 1.46x10^-02^ |
|  | rs34320000 | 7.87x10^-02^ | 1.59x10^-01^ | 3.24x10^-02^ |
|  | rs5957596 | 1.14x10^-01^ | 2.01x10^-01^ | 3.92x10^-02^ |
|  | rs10217856 | 4.06x10^-02^ | 6.84x10^-02^ | 1.31x10^-02^ |
|  | rs5956287 | 2.71x10^-01^ | 2.51x10^-01^ | 4.63x10^-02^ |
|  | rs12834182 | 2.71x10^-01^ | 2.51x10^-01^ | 4.63x10^-02^ |
|  | rs1121546 | 1.27x10^-02^ | 2.79x10^-02^ | 6.42x10^-03^ |
|  | rs5957620 | 2.17x10^-02^ | 3.51x10^-02^ | 6.95x10^-03^ |
|  | rs9887587 | 2.06x10^-02^ | 2.97x10^-02^ | 5.71x10^-03^ |
|  | rs150986507 | 1.66x10^-02^ | 3.31x10^-02^ | 6.63x10^-03^ |
|  | rs12839589 | 5.00x10^-02^ | 8.37x10^-02^ | 1.73x10^-02^ |
|  | rs33977652 | 2.56x10^-01^ | 2.66x10^-01^ | 4.86x10^-02^ |
|  | rs138347087 | 2.26x10^-01^ | 2.39x10^-01^ | 4.38x10^-02^ |
|  | rs35046609 | 2.91x10^-02^ | 4.81x10^-02^ | 9.59x10^-03^ |
|  | rs60669023 | 2.83x10^-01^ | 2.43x10^-01^ | 4.50x10^-02^ |
|  | rs16996189 | 2.97x10^-01^ | 2.60x10^-01^ | 4.82x10^-02^ |
|  | rs5957651 | 2.98x10^-02^ | 5.49x10^-02^ | 1.11x10^-02^ |
|  | rs148797601 | 6.88x10^-03^ | 1.94x10^-02^ | 4.33x10^-03^ |
|  | rs148097246 | 7.20x10^-03^ | 2.04x10^-02^ | 4.58x10^-03^ |
|  |  |  |  |  |
| **WT2 AS** | rs7057428 | 2.04x10^-02^ | 1.0 | 1.0 |
|  | rs5977756 | 7.21x10^-03^ | 9.05x10^-02^ | 2.78x10^-01^ |
|  |  |  |  |  |
| **WT2 UC** | rs5916435 | 1.80x10^-03^ | 1.22x10^-03^ | 2.19x10^-04^ |
|  | rs5973636 | 1.15x10^-01^ | 4.76x10^-02^ | 8.74x10^-03^ |
|  | rs6610386 | 3.72x10^-02^ | 3.76x10^-02^ | 2.24x10^-02^ |
|  | rs59269143 | 2.36x10^-02^ | 2.26x10^-02^ | 1.19x10^-02^ |
|  | rs5963157 | 2.73x10^-02^ | 2.66x10^-02^ | 1.47x10^-02^ |
|  | rs7060409 | 7.16x10^-03^ | 6.50x10^-03^ | 2.64x10^-03^ |
|  | rs62626573 | 1.81x10^-02^ | 1.63x10^-02^ | 4.70x10^-03^ |
|  | rs35764713 | 6.96x10^-07^ | 3.31x10^-09^ | 7.72x10^-10^ |
|  | rs5969304 | 7.58x10^-05^ | 1.0 | 1.0 |
|  | rs6643227 | 3.05x10^-03^ | 1.0 | 1.0 |
|  | rs6655215 | 2.04x10^-04^ | 5.75x10^-03^ | 1.03x10^-01^ |
|  | rs12008980 | 8.74x10^-05^ | 5.73x10^-02^ | 5.85x10^-01^ |
