# Supplementary material for: Accounting for eXentricities: Analysis of the X Chromosome in GWAS Reveals X-Linked Genes Implicated in Autoimmune Diseases
Source: PLoS One. 2014 Dec 5;9(12):e113684. doi: 10.1371/journal.pone.0113684 (PMC4257614; doi:10.1371/journal.pone.0113684)
Supplement: Table S3 — All genes with either truncated tail or truncated product P<1×10^−3^ for the FMF.comb and the FMS.comb tests. (DOC) [file pone.0113684.s008.doc]

| **FM_F.comb_** | | | |
| --- | --- | --- | --- |
| **Dataset** | **Gene symbol** | **Truncated tail p-value** | **Truncated product p-value** |
| ALS Finland | TAF7L | 0.000389 | 0.0018 |
| ALS Finland | MAGEE2 | 0.00028 | 0.0012 |
| ALS Finland | NAP1L2 | 0.00091 | 0.00034 |
| ALS Finland | TTC3P1 | 0.000859 | 0.0013 |
| ALS Finland | ZDHHC15 | 0.000413 | 0.0089 |
| CASP | NLGN4X | 0.000887 | 0.0166 |
| Celiac disease CIDR | CENPI | 0.0029 | 0.000523 |
| Vitiligo GWAS1 | PPP1R3F | 0.000114 | 0.000496 |
| Vitiligo GWAS1 | LINC00632 | 0.0057 | 0.000772 |
| Vitiligo GWAS1 | FOXP3 | 0.000698 | 0.0015 |
| Vitiligo GWAS1 | BEND2 | 0.0018 | 0.000079 |
| Vitiligo GWAS1 | CENPI | 0.000155 | 0.0026 |
| Vitiligo GWAS2 | IL13RA2 | 0.0021 | 0.000758 |
| Vitiligo GWAS2 | MCF2 | 0.00017 | 0.000576 |
| CD WT1 | CD40LG | 0.009 | 0.000322 |
| CD WT1 | LINC00892 | 0.0013 | 0.000088 |
| T2D WT1 | MAGEC1 | 0.0275 | 0.000181 |
| UC WT2 | CASK | 0.000138 | 0.0215 |
| UC WT2 | PRPS1 | 0.000133 | 0.000194 |
| UC WT2 | PAGE2B | 0.0039 | 0.000012 |
| UC WT2 | SPANXN5 | 0.00091 | 0.0013 |
| MS WT2 | MAGEE1 | 0.000706 | 0.0023 |
| **FM_S.Comb_** | | | |
| ALS Finland | TAF7L | 0.000547 | 0.000644 |
| ALS Finland | NAP1L2 | 0.00057 | 0.000115 |
| ALS Finland | ITM2A | 0.000843 | 0.000307 |
| ALS Finland | CENPI | 0.001271 | 0.000175 |
| ALS Finland | TMEM35 | 0.002775 | 0.000345 |
| CASP | MIR505 | <1x10^-6^ | 0.001932 |
| CASP | DCX | 0.000757 | 0.00608 |
| Celiac CIDR | IQSEC2 | 0.00053 | 0.00071 |
| CD WT1 | Y RNA | <1x10^-6^ | 0.000052 |
| CD WT1 | LINC00892 | 0.001739 | 0.000529 |
| UC WT2 | PRPS1 | 0.000005 | 0.000005 |
| UC WT2 | CASK | 0.000157 | 0.021124 |
| UC WT2 | GPR82 | 0.000209 | 0.001885 |
| UC WT2 | GPR34 | 0.000262 | 0.000162 |
| UC WT2 | PAGE2B | 0.000482 | 0.000002 |
| UC WT2 | NAP1L6 | 0.001192 | 0.000429 |
| MS Case Control | RP11-265P11.2 | 0.00303 | 0.000855 |
| Vitiligo GWAS1 | PPP1R3F | 0.000006 | 0.000076 |
| Vitiligo GWAS1 | FOXP3 | 0.000022 | 0.000149 |
| Vitiligo GWAS1 | XRCC6P5 | 0.000081 | 0.001846 |
| Vitiligo GWAS1 | HUWE1 | 0.000362 | 0.001298 |
| Vitiligo GWAS1 | GAGE12H | 0.000634 | 0.000634 |
| Vitiligo GWAS1 | GAGE10 | 0.001848 | 0.000266 |
| Vitiligo GWAS2 | MCF2 | 0.000078 | 0.000131 |
| Vitiligo GWAS2 | IL13RA2 | 0.000942 | 0.000354 |
| Vitiligo GWAS2 | RBMXL3 | 0.002653 | 0.000321 |
| T2D GENEVA | ZCCHC12 | 0.001209 | 0.000653 |
| T2D GENEVA | SNORA35 | 0.002123 | 0.000454 |
| T2D GENEVA | IL13RA1 | 0.00635 | 0.000859 |
| T2D WT1 | MAGEC1 | 0.026251 | 0.000068 |
| T1D WT1 | ARX | 0.000192 | 0.000489 |
| T1D WT1 | SRPK3 | 0.000469 | 0.008982 |
| T1D WT1 | PLXNB3 | 0.000487 | 0.007309 |
| T1D WT1 | RNU6-98P | 0.000803 | 0.001921 |
