# Supplementary material for: Accounting for eXentricities: Analysis of the X Chromosome in GWAS Reveals X-Linked Genes Implicated in Autoimmune Diseases
Source: PLoS One. 2014 Dec 5;9(12):e113684. doi: 10.1371/journal.pone.0113684 (PMC4257614; doi:10.1371/journal.pone.0113684)
Supplement: Table S4 — All genes with either truncated tail or truncated product P<1×10^−3^ for the FM02 test. (DOC) [file pone.0113684.s009.doc]

| **Dataset** | **Gene symbol** | **Truncated tail p-value** | **Truncated product p-value** |
| --- | --- | --- | --- |
| ALS Finland | TAF7L | 0.000126 | 0.000332 |
| ALS Finland | NAP1L2 | 0.000451 | 0.000038 |
| ALS Finland | ITM2A | 0.0021 | 0.00041 |
| CASP | PGRMC1 | <1x10^-6^ | 0.0046 |
| CASP | ATP11C | 0.000011 | 0.0092 |
| CASP | DCX | 0.000752 | 0.0048 |
| CASP | MIR505 | <1x10^-6^ | 0.0039 |
| MS case control | FANCB | 0.000052 | 0.0013 |
| MS case control | RP11-265P11.2 | 0.0025 | 0.000423 |
| Vitiligo GWAS1 | PPP1R3F | 0.000066 | 0.000139 |
| Vitiligo GWAS1 | HUWE1 | 0.000822 | 0.0027 |
| Vitiligo GWAS1 | LINC00632 | 0.0137 | 0.000453 |
| Vitiligo GWAS1 | FOXP3 | 0.000111 | 0.000276 |
| Vitiligo GWAS1 | GAGE10 | 0.0016 | 0.000403 |
| Vitiligo GWAS1 | CENPI | 0.000217 | 0.001 |
| Vitiligo GWAS1 | MPC1L | <1x10^-6^ | <1x10^-6^ |
| Vitiligo GWAS1 | NAA10 | 0.00087 | 0.0028 |
| Vitiligo GWAS2 | IL13RA2 | 0.001014 | 0.000526 |
| Vitiligo GWAS2 | MCF2 | 0.000224 | 0.000559 |
| Vitiligo GWAS2 | RBMXL3 | 0.0019 | 0.000418 |
| GENEVA T2D | RP4-562J12.2 | 0.000489 | 0.0013 |
| CD WT1 | ARHGEF6 | 0.0017 | 0.000366 |
| CD WT1 | CD40LG | 0.0123 | 0.000223 |
| CD WT1 | LINC00892 | 0.001572 | 0.000048 |
| T1D WT1 | SRPK3 | 0.000327 | 0.0071 |
| T1D WT1 | ARX | 0.000837 | 0.000565 |
| T1D WT1 | RNU6-98P | 0.000716 | 0.0018 |
| T1D WT1 | PLXNB3 | 0.000522 | 0.0076 |
| T2D WT1 | MAGEC1 | 0.0264 | 0.000534 |
| T2D WT1 | SASH3 | <1x10^-6^ | <1x10^-6^ |
| T2D WT1 | DUSP9 | 0.0022 | 0.000553 |
| UC WT2 | CASK | 0.000357 | 0.0199 |
| UC WT2 | PRPS1 | 0.000003 | 0.00001 |
| UC WT2 | NAP1L6 | 0.001063 | 0.000057 |
| UC WT2 | PAGE2B | 0.012 | 0.000072 |
| UC WT2 | GPR34 | 0.0011 | 0.00061 |
