# Supplementary material for: Accounting for eXentricities: Analysis of the X Chromosome in GWAS Reveals X-Linked Genes Implicated in Autoimmune Diseases
Source: PLoS One. 2014 Dec 5;9(12):e113684. doi: 10.1371/journal.pone.0113684 (PMC4257614; doi:10.1371/journal.pone.0113684)
Supplement: Table S5 — CENPI association p-values for the FMF.comb test across the 16 datasets. (DOC) [file pone.0113684.s010.doc]

| **Dataset** | **p-value (tail, product)** |
| --- | --- |
| ALS Finland | 1.10x10^-2^, 1.00x10^-3^ |
| ALS Irish | 2.70x10^-2^, 1.60x10^-2^ |
| CASP | 0.91, 0.64 |
| CIDR Celiac | 2.9x10^-3^, 5.23x10^‑4^ |
| NIDDK CD | 0.17, 0.16 |
| MS case control | 0.91, 0.38 |
| Vitiligo GWAS1 | 1.55x10^-4^, 2.6x10^-3^ |
| Vitiligo GWAS2 | 0.827, 0.65 |
| Geneva T2D | 0.17, 0.19 |
| WT1 CD | 0.83, 0.20 |
| WT1 T1D | 0.85, 0.49 |
| WT1 RA | 0.83, 0.29 |
| WT1 T2D | 0.93, 0.54 |
| WT2 UC | 0.88, 0.45 |
| WT2 MS | 0.81, 0.67 |
| WT2 AS | 0.11, 0.11 |
