# Supplementary material for: Accounting for eXentricities: Analysis of the X Chromosome in GWAS Reveals X-Linked Genes Implicated in Autoimmune Diseases
Source: PLoS One. 2014 Dec 5;9(12):e113684. doi: 10.1371/journal.pone.0113684 (PMC4257614; doi:10.1371/journal.pone.0113684)
Supplement: Table S6 — All genes with either the truncated tail or truncated product P<1×10^−3^ for the sex difference test. (DOC) [file pone.0113684.s011.doc]

| **Dataset** | **Gene symbol** | **Truncated tail p-value** | **Truncated product p-value** |
| --- | --- | --- | --- |
| ALS Finland | MAGEE2 | 6.50x10^-4^ | 1.93x10^-3^ |
| ALS Finland | NDP | 1.41x10^-3^ | 9.34x10^-4^ |
| CASP | NLGN4X | 2.34x10^-4^ | 0.017 |
| CIDR Celiac | CENPI | 4.42x10^-3^ | 2.08x10^-4^ |
| CD WT1 | C1GALT1C1 | 1.97x10^-3^ | 2.63x10^-4^ |
| UC WT2 | SPANXN5 | 2.72x10^-4^ | 3.45x10^-4^ |
| UC WT2 | XAGE5 | 2.21x10^-3^ | 3.45x10^-4^ |
| MS case control | ZNF449 | 7.22x10^-4^ | 3.11x10^-3^ |
| MS case control | BMX | 9.91x10^-4^ | 2.49x10^-3^ |
| Vitiligo GWAS1 | BEND2 | 3.99x10^-3^ | 1.28x10^-4^ |
| Vitiligo GWAS2 | MCF2 | 7.00x10^-4^ | 1.93x10^‑3^ |
| T2D GENEVA | EFHC2 | 6.09x10^-4^ | 1.1x10^-3^ |
| T2D WT1 | SASH3 | <1x10^-6^ | <1x10^-6^ |
| T2D WT1 | CSTF2 | 1.63x10^-3^ | 8.17x10^-4^ |
| T2D WT1 | SNORA9 | 1.63x10^-3^ | 8.43x10^-4^ |
| T2D WT1 | SYTL4 | 2.27x10^-3^ | 3.27x10^-4^ |
| RA WT1 | MIR320D2 | 8.69x10^-3^ | 5.68x10^-4^ |
