# Supplementary material for: Accounting for eXentricities: Analysis of the X Chromosome in GWAS Reveals X-Linked Genes Implicated in Autoimmune Diseases
Source: PLoS One. 2014 Dec 5;9(12):e113684. doi: 10.1371/journal.pone.0113684 (PMC4257614; doi:10.1371/journal.pone.0113684)
Supplement: Table S7 — All p-values for all gene sets and all datasets are listed. Those with P<0.05 are highlighted in Table 4 in the main text. (DOC) [file pone.0113684.s012.doc]

| **XY homologs gene set** | | | | | | |
| --- | --- | --- | --- | --- | --- | --- |
| **Dataset** | **FM_02_** | | **FM_F.comb_** | | **FM_S.comb_** | |
|  | **Tail** | **Product** | **Tail** | **Product** | **Tail** | **Product** |
| ALS Finland | 0.672 | 0.608 | 0.448 | 0.707 | 0.653 | 0.461 |
| ALS Irish | 0.967 | 0.953 | 0.282 | 0.19 | 0.958 | 0.959 |
| Psoriasis CASP | 0.779 | 0.826 | 0.0088 | 0.123 | 0.429 | 0.318 |
| Celiac Disease CIDR | 0.0546 | 0.12 | 0.0467 | 0.146 | 0.107 | 0.16 |
| T2D GENEVA | 0.256 | 0.202 | 0.203 | 0.198 | 0.257 | 0.184 |
| CD NIDDK | 0.017 | 0.117 | 0.129 | 0.151 | 0.0701 | 0.108 |
| MS Case Control | 0.556 | 0.442 | 0.651 | 0.476 | 0.202 | 0.188 |
| Vitiligo GWAS1 | 0.0329 | 0.067 | 0.0063 | 0.0675 | 0.15 | 0.187 |
| Vitiligo GWAS2 | 0.144 | 0.15 | 0.0346 | 0.117 | 0.189 | 0.16 |
| AS WT2 | 0.129 | 0.145 | 0.42 | 0.513 | 0.0958 | 0.142 |
| MS WT2 | 0.1031 | 0.158 | 0.159 | 0.245 | 0.522 | 0.333 |
| UC WT2 | 0.418 | 0.328 | 0.832 | 0.604 | 0.741 | 0.577 |
| CD WT1 | 0.0197 | 0.0859 | 0.141 | 0.111 | 0.0546 | 0.0865 |
| RA WT1 | 0.177 | 0.213 | 0.391 | 0.408 | 0.582 | 0.406 |
| T1D WT1 | 0.206 | 0.201 | 0.105 | 0.134 | 0.0402 | 0.0843 |
| T2D WT1 | 0.458 | 0.482 | 0.866 | 0.752 | 0.685 | 0.597 |
| **Panther immune gene set** | | | | | | |
| ALS Finland | 0.866 | 0.898 | 0.789 | 0.879 | 0.964 | 0.915 |
| ALS Irish | 0.986 | 0.985 | 1 | 1 | 1 | 0.939 |
| Psoriasis CASP | 0.996 | 0.968 | 0.908 | 0.94 | 0.934 | 0.984 |
| Celiac Disease CIDR | 0.793 | 0.797 | 0.808 | 0.83 | 0.736 | 0.83 |
| T2D GENEVA | 0.0132 | 0.893 | 0.0233 | 0.875 | 0.0073 | 0.913 |
| CD NIDDK | 0.585 | 0.895 | 0.814 | 0.933 | 0.738 | 0.9 |
| MS Case Control | 0.962 | 0.931 | 0.849 | 0.969 | 0.976 | 0.697 |
| Vitiligo GWAS1 | 0.0154 | 0.697 | 0.0387 | 0.683 | 0.0081 | 0.683 |
| Vitiligo GWAS2 | 0.0142 | 0.79 | 0.0448 | 0.807 | 0.0127 | 0.813 |
| AS WT2 | 0.177 | 0.863 | 0.642 | 0.876 | 0.262 | 0.862 |
| MS WT2 | 0.38 | 0.693 | 0.475 | 0.735 | 0.54 | 0.707 |
| UC WT2 | 0.1 | 0.825 | 0.252 | 0.819 | 0.0683 | 0.806 |
| CD WT1 | 0.135 | 0.458 | 0.329 | 0.538 | 0.114 | 0.466 |
| RA WT1 | 0.361 | 0.525 | 0.531 | 0.609 | 0.865 | 0.642 |
| T1D WT1 | 0.33 | 0.53 | 0.574 | 0.707 | 0.524 | 0.498 |
| T2D WT1 | 0.857 | 0.897 | 0.787 | 0.892 | 0.914 | 0.963 |
| **KEGG/GO immune gene set** | | | | | | |
| ALS Finland | 0.131 | 0.153 | 0.679 | 0.522 | 0.136 | 0.135 |
| ALS Irish | 0.965 | 0.97 | 0.987 | 0.999 | 0.951 | 0.796 |
| Psoriasis CASP | 0.884 | 0.734 | 0.501 | 0.529 | 0.774 | 0.512 |
| Celiac Disease CIDR | 0.496 | 0.467 | 0.334 | 0.527 | 0.239 | 0.416 |
| T2D GENEVA | 0.223 | 0.221 | 0.147 | 0.132 | 0.878 | 0.658 |
| CD NIDDK | 0.313 | 0.54 | 0.414 | 0.552 | 0.397 | 0.522 |
| MS Case Control | 0.557 | 0.894 | 0.33 | 0.467 | 0.803 | 0.881 |
| Vitiligo GWAS1 | 0.000453 | 0.0613 | 0.002 | 0.0621 | 0.000164 | 0.063 |
| Vitiligo GWAS2 | 0.235 | 0.33 | 0.423 | 0.569 | 0.236 | 0.38 |
| AS WT2 | 0.878 | 0.675 | 0.765 | 0.664 | 0.914 | 0.802 |
| MS WT2 | 0.902 | 0.875 | 0.73 | 0.853 | 0.541 | 0.684 |
| UC WT2 | 0.332 | 0.314 | 0.701 | 0.68 | 0.566 | 0.431 |
| CD WT1 | 0.224 | 0.226 | 0.3 | 0.512 | 0.297 | 0.471 |
| RA WT1 | 0.927 | 0.996 | 0.653 | 0.956 | 0.944 | 0.91 |
| T1D WT1 | 0.241 | 0.605 | 0.274 | 0.38 | 0.276 | 0.397 |
| T2D WT1 | 0.651 | 0.788 | 0.525 | 0.849 | 0.671 | 0.769 |
