# Supplementary material for: Accounting for eXentricities: Analysis of the X Chromosome in GWAS Reveals X-Linked Genes Implicated in Autoimmune Diseases
Source: PLoS One. 2014 Dec 5;9(12):e113684. doi: 10.1371/journal.pone.0113684 (PMC4257614; doi:10.1371/journal.pone.0113684)
Supplement: Table S8 — Pairs of X-linked genes that are significantly co-expressed. Presented are pairs of genes that are significantly co-expressed, after multiple hypothesis correction, along with the squared Spearman's correlation coefficient (r2) and p-value of a Spearman's rank correlation test (Materials and Methods). (DOCX) [file pone.0113684.s013.docx]

| **Gene_1_** | **Gene_2_** | **r^2^** | **P-value** |
| --- | --- | --- | --- |
| *ARHGEF6* | *EFHC2* | 0.043264 | 4.44x10^-10^ |
| *ARHGEF6* | *IL13RA1* | 0.071289 | 7.46x10^-16^ |
| *ARHGEF6* | *PPP1R3F* | 0.091204 | 4.41x10^-20^ |
| *BEND2* | *EFHC2* | 0.034225 | 3.39x10^-8^ |
| *C1GALT1C1* | *EFHC2* | 0.201601 | 6.80x10^-45^ |
| *C1GALT1C1* | *FANCB* | 0.031684 | 1.12x10^-7^ |
| *C1GALT1C1* | *IL13RA1* | 0.048841 | 3.31x10^-11^ |
| *C1GALT1C1* | *ITM2A* | 0.051076 | 1.27x10^-11^ |
| *C1GALT1C1* | *PPP1R3F* | 0.077284 | 4.57x10^-17^ |
| *EFHC2* | *FANCB* | 0.036864 | 8.49x10^-9^ |
| *EFHC2* | *IL13RA1* | 0.04 | 2.17x10^-9^ |
| *EFHC2* | *ITM2A* | 0.084681 | 1.13x10^-18^ |
| *EFHC2* | *PPP1R3F* | 0.246016 | 6.03x10^-56^ |
| *FANCB* | *PPP1R3F* | 0.033489 | 4.33x10^-8^ |
| *ITM2A* | *PPP1R3F* | 0.076176 | 7.89x10^-17^ |
| *NLGN4X* | *TMEM35* | 0.037249 | 8.31x10^-9^ |
