# Supplementary material for: Accounting for eXentricities: Analysis of the X Chromosome in GWAS Reveals X-Linked Genes Implicated in Autoimmune Diseases
Source: PLoS One. 2014 Dec 5;9(12):e113684. doi: 10.1371/journal.pone.0113684 (PMC4257614; doi:10.1371/journal.pone.0113684)
Supplement: Table S9 — List of genes in the KEGG/GO immune gene set. (DOC) [file pone.0113684.s014.doc]

| **Gene symbol** |
| --- |
| OTUD5 |
| TLR8 |
| CFP |
| RNF128 |
| PRKX |
| APLN |
| BTK |
| IL3RA |
| IKBKG |
| IRAK1 |
| CD40LG |
| SH2D1A |
| XIAP |
| NOX1 |
| CXCR3 |
| IL2RG |
| EDA |
| FOXP3 |
| WAS |
| CYBB |
| TAB3 |
| TLR7 |
| CD99 |
| DDX3X |
| CSF2RA |
| IL9R |
| BCAP31 |
