# Supplementary material for: Accounting for eXentricities: Analysis of the X Chromosome in GWAS Reveals X-Linked Genes Implicated in Autoimmune Diseases
Source: PLoS One. 2014 Dec 5;9(12):e113684. doi: 10.1371/journal.pone.0113684 (PMC4257614; doi:10.1371/journal.pone.0113684)
Supplement: Text S1 — Supplementary information detailing single-SNP association analysis and power calculations for gene-based tests. (DOCX) [file pone.0113684.s015.docx]

**SUPPLEMENTARY INFORMATION**

Supplementary Text 2

SNP-level results 2

Power calculations 3

Figures 6

# Supplementary Text

## SNP-level results

The single nucleotide polymorphism (SNP) association analysis (see “Single-SNP association analysis” in the Materials and Methods of the main text), including imputed SNPs, identified 42 SNPs significantly associated with their respective disease following a conservative Bonferroni correction for the number of tests (Supplementary Fig. S2a, Table S2). Of these, 14 SNPs in the same locus form a clear peak (Supplementary Fig. S2b) in their association with vitiligo (Vitiligo GWAS1 dataset). Vitiligo is a common autoimmune disorder in which the destruction of melanocytes (pigment producing cells located in the basal epidermis) results in depigmented skin. The associated locus is 17 kilobases (kb) away from a weakly expressed retrotransposed gene (retro-*HSPA8*) that is of 98% similarity to its parent gene, *HSPA8*, on chromosome 11. *HSPA8* encodes a member of the heat shock protein 70 family and functions as a chaperone to bind nascent polypeptides and enable correct folding. Heat shock proteins have been previously implicated in autoimmune disease [[1-5](#_ENREF_1)]. In particular, a role for inducible heat shock protein 70 has been suggested in vitiligo [[6-8](#_ENREF_6)]. Though this region did not replicate in our second vitiligo dataset, the biological relevance of this region warrants further investigation in a larger, better powered replication study. Another clear association peak was observed for the Wellcome Trust Case Control Consortium 2 ulcerative colitis (WT2 UC) (Supplementary Fig. S2c) for intronic variants of *BCOR*. *BCOR* encodes a co-repressor of *BCL-6*, which regulates apoptosis [[9](#_ENREF_9)]. However, none of these candidate associations replicated in other GWAS datasets for the same or related disease, possibly due to small sample sizes and thus insufficient power (Table 1).

##

## Power calculations

We used simulations to test the power of each of our single-SNP test statistics (FM_02_, FM_F.comb_, FM_S.comb_ and the difference in effect size test; Materials and Methods). We first randomly assigned genotypes for *m* males and *f* females with a certain minor allele frequency (MAF). As we are interested in X-linked loci, males are simulated as hemizygous.

For each individual, we simulated a quantitative phenotype (*q*_i_) assuming an effect size $\beta$*.* We note that this effect size is not equivalent to that generally considered in GWAS as we do not take into account prevalence. For females:$q_{i}=\left[\begin{matrix} N(0,1) if AA \\ \frac{\beta}{2}+N(0,1) if Aa \\ \beta+N(0,1) if aa \end{matrix}\right.$, where **AA** represents female homozygotes for the major allele, **aa** homozygotes for the minor allele, and **Aa** heterozygotes. In males, when assigning the equivalent phenotype to female homozygotes, we have: $q_{i}=\left[\begin{matrix} N(0,1) if A \\ \\ \beta+N(0,1) if a \end{matrix}\right.$. In the following we also consider scenarios with different $\beta$between males and females. We next transformed these quantitative phenotypes into probabilities of being a case assuming a normal distribution (thus individuals with a larger phenotype will be more likely to be assigned as a case) and randomly assign case or control status to each individual based on this probability. Due to the probabilistic assignment of case or control status, we first simulated a larger population and then subsampled the desired number of cases and controls from this larger pool.

In another set of simulations, we considered female phenotypes assuming a scenario of complete X-inactivation. Under this scenario, female heterozygotes are assigned quantitative phenotypes as if they were either homozygote, with equal probability. Thus, under X-inactivation, a heterozygous female has the following phenotype (with probability *p*): $q_{i}=\left[\begin{matrix} N(0,1), p=0.5 \\ \\ \beta+N(0,1), p=0.5 \end{matrix}\right.$. We followed with assigning case or control status based on these revised *q_i_* as above.

We tested the power of the 4 statistical tests applied in this study across different effect sizes ($\beta$for males and $\beta$for females) and sample sizes (*m* and *f*). We simulated for each scenario 500 genotype-phenotype combinations and counted for each test the fraction of these simulations that reached P < 1x10^-6^ (equivalent to X-chromosome-wide significance following Bonferroni correction).

Applying these tests to our simulated data under the various conditions, we found that overall the FM_02_ test was the most powerful test when male and female effect size was the same (Figures A1-A4). Yet, even with the FM_02_ test, power was limited with the sample sizes present in the majority of the empirical data analyzed in this study, e.g. only 53% detection of a variant with an overall sample size of 2000 and an effect size of 0.5 (Figure A1). Across the two sex-stratified tests, FM_F.comb_ performs better than FM_S.comb_ when sample sizes are equal between the sexes (Figures A1-A2), while FM_S.comb_—which weighs the two sex-specific tests by the sample size of each sex—is more powerful when sample size is sex-biased (Figure A3). As expected, power decreases for each of the 3 tests of association when effect size varies between males and females (Figure A4). In addition, the sex-stratified FM_F.comb_ test generally becomes more powerful than FM_02_. The test for differential effect size has some power in these scenarios, e.g. ~41% with effect sizes of 0.5 and 0.1 in males and females, respectively, and a sample size of 5000 (Figure A4). In these scenarios it is even more powerful than one of the sex-stratified tests of association (FM_S.comb_). Many more scenarios will need to be simulated to fully characterize the utility of the different tests in different scenarios.

# Figures

**
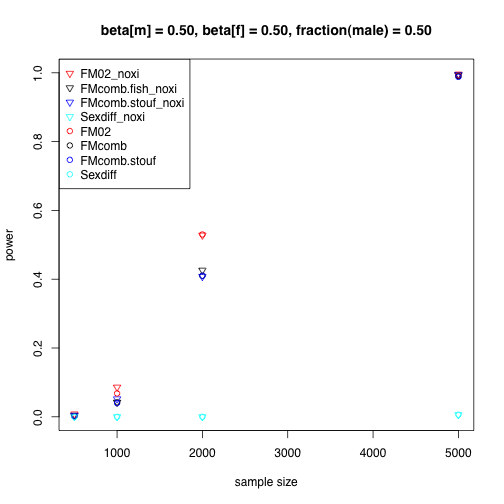
Figure A1. Power calculations assuming an equal effect size of 0.5.** For a sample size of 500, 1000, 2000 and 5000, equally distributed between male and female cases and controls. Presented is the fraction of 500 simulations that result in a P < 1x10^-6^ for each test. Scenarios assuming no X-inactivation (_*noxi)* are denoted in triangles, whereas scenarios assuming full X-inactivation are represented in circles. The FM_02_ (*FM02*), FM_F.comb_ (*FMcomb.fish*), FM_S.comb_ (*FMcomb.stouf*), and the difference in effect size between sex (*Sexdiff*) tests are plotted in red, black, blue and cyan, respectively. All tests, with the exception of the sex difference test, are well powered to detect variants given a large enough sample size. We note that since effect sizes are equal between the sexes, the null is met for the *Sexdiff* test, thus the figure does not denote power for this test, rather proportion of false positives, which is effectively 0.


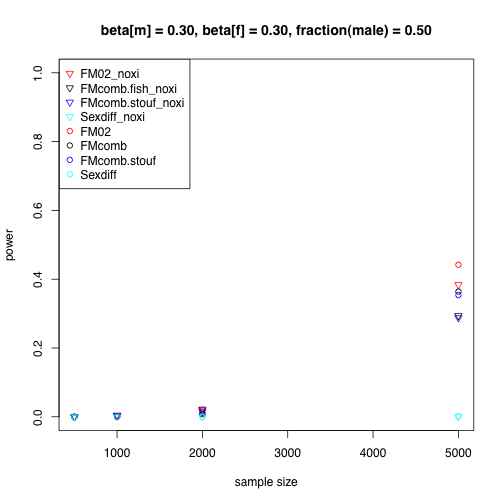


**Figure A2. Power calculations for equal effect size of 0.3.** This figure mirrors Figure A1 except simulations assume causal variants with an effect size of 0.3.


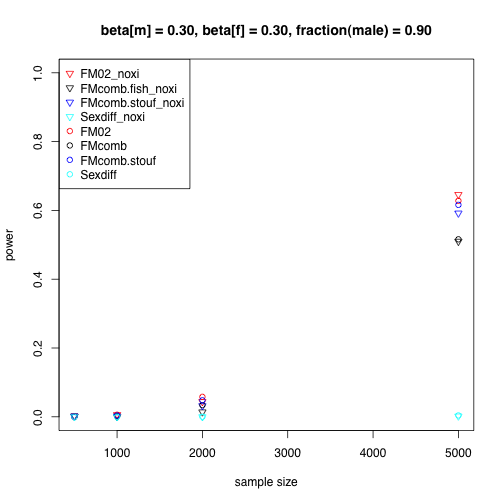
 **Figure A3. Power calculations for equal effect size of 0.5 and unequal sample size between males and females.** This figure mirrors Figure A1 except that sample size is different between males and females, with males constituting 90% of all samples. A reduction in power is observed as compared to the scenario presented in Figure A1. Across the sex-stratified tests, *FMcomb.stouf*, which weighs the two sex-specific tests based on the sample size in each sex, outperforms *FMcomb.fish*.


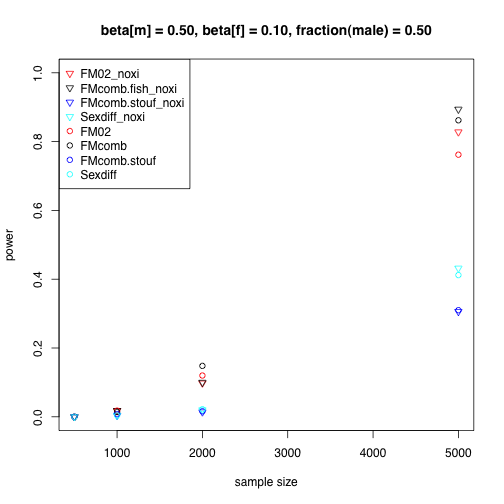


**Figure A4. Power calculations for unequal effect size between males and females.** This figure mirrors Figure A1 except for differing effect size between males (0.5) and females (0.1). The sex-stratified FM_F.comb_ test is slightly better powered than the FM_02_ test, while the opposite has been observed in scenarios with equal effect sizes (Figures A1-A3).

#

**REFERENCES**

1. Routsias JG, Tzioufas AG (2006) The role of chaperone proteins in autoimmunity. Annals of the New York Academy of Sciences 1088: 52-64.

2. Winfield JB, Jarjour WN (1991) Stress proteins, autoimmunity, and autoimmune disease. Current Topics in Microbiology and Immunology 167: 161-189.

3. Rauch SD, San Martin JE, Moscicki RA, Bloch KJ (1995) Serum antibodies against heat shock protein 70 in Meniere's disease. The American journal of otology 16: 648-652.

4. Naumann A, Hempel JM, Schorn K (2001) [Detection of humoral immune response to inner ear proteins in patients with sensorineural hearing loss]. Laryngo- rhino- otologie 80: 237-244.

5. Ludwig D, Stahl M, Ibrahim ET, Wenzel BE, Drabicki D, et al. (1999) Enhanced intestinal expression of heat shock protein 70 in patients with inflammatory bowel diseases. Digestive Diseases and Sciences 44: 1440-1447.

6. Mosenson JA, Zloza A, Klarquist J, Barfuss AJ, Guevara-Patino JA, et al. (2012) HSP70i is a critical component of the immune response leading to vitiligo. Pigment cell & melanoma research 25: 88-98.

7. Mosenson JA, Eby JM, Hernandez C, Le Poole IC (2013) A central role for inducible heat-shock protein 70 in autoimmune vitiligo. Experimental dermatology 22: 566-569.

8. Abdou AG, Maraee AH, Reyad W (2013) Immunohistochemical expression of heat shock protein 70 in vitiligo. Annals of diagnostic pathology 17: 245-249.

9. Huynh KD, Fischle W, Verdin E, Bardwell VJ (2000) BCoR, a novel corepressor involved in BCL-6 repression. Genes & Development 14: 1810-1823.
